# Psychosocial interventions for managing occupational stress and burnout among medical doctors: a systematic review

**DOI:** 10.1186/s13643-017-0526-3

**Published:** 2017-07-17

**Authors:** Bonnie A. Clough, Sonja March, Raymond J. Chan, Leanne M. Casey, Rachel Phillips, Michael J. Ireland

**Affiliations:** 1 School of Psychology and Counselling, Institute for Resilient Regions, University of Southern Queensland, 37 Sinnathamby Boulevard, Springfield Central, QLD 4300 Australia; 2 Cancer Nursing Professorial Precinct, Queensland University of Technology, Brisbane, QLD Australia; 3 Royal Brisbane and Women’s Hospital, Brisbane, QLD Australia; 4 School of Applied Psychology, Menzies Health Institute Queensland, Griffith University, Brisbane, QLD Australia; 5 West Moreton Hospital and Health Service, Queensland Health, Ipswich, QLD Australia; 6 Current address: School of Applied Psychology, Menzies Health Institute Queensland, Griffith University, Gold Coast Campus, 58 Parklands Drive, Southport, QLD 4215 Australia

**Keywords:** Stress, Burnout, Doctors, Physician, Medical practitioner

## Abstract

**Background:**

Occupational stress and burnout are highly prevalent among medical doctors and can have adverse effects on patient, doctor, and organisational outcomes. The purpose of the current study was to review and evaluate evidence on psychosocial interventions aimed at reducing occupational stress and burnout among medical doctors.

**Method:**

A systematic review was conducted for original research articles reporting on psychosocial interventions targeting occupational stress or burnout among medical doctors, published in the English language, and with data collected at a minimum of two time points. Searches were conducted across five electronic databases, as well as by manual search of Google Scholar. Data was extracted relating to study characteristics and outcomes, quality and rigour, as well as modes of delivery and engagement. Studies were appraised using the Strength of Recommendation Taxonomy (SORT) and Critical Appraisal Skills Programme (CASP).

**Results:**

Twenty-three articles were reviewed, which reported on interventions utilising cognitive-behavioural, relaxation, and supportive discussion strategies. Only 12 studies allowed estimation of pre- to post-intervention effects. Cognitive behavioural interventions demonstrated the strongest evidence, particularly for reducing stress. Some evidence was identified to support the efficacy of relaxation-based approaches, but no such evidence was found for the efficacy of discussion-based interventions, such as Balint groups. There was a lack of quality among reviewed studies, with no studies receiving a quality rating of 1, and the overall body of evidence being rated as level B, according to the SORT. Effect sizes were not pooled due to a lack of quality among the study sample.

**Conclusion:**

This review found that despite increased scientific attention, the quality of research examining the benefits of psychosocial/behavioural interventions for occupational stress and burnout in medical doctors remains low. Despite this, interventions focused on cognitive and behavioural principles appear to show promise in reducing doctor stress and burnout. Limitations of the current review include a lack of risk of bias assessment or pooling of analyses. Recommendations for improving the quality of research in this area, as well as implications of the current body of evidence are discussed.

**Systematic review registration:**

PROSPERO CRD42016032595

**Electronic supplementary material:**

The online version of this article (doi:10.1186/s13643-017-0526-3) contains supplementary material, which is available to authorized users.

The prevalence of burnout has been found to be as high as 75% among doctors [1, 2], with the highest rates often observed among junior doctors and those working at the front line of patient care [3, 4]. Among doctors, occupational stress and burnout have been associated with poorer quality of personal relationships, individual wellbeing, and patient care [5–8]. Although sometimes comorbid with mental health issues such as anxiety, depression, and substance use [9–11], burnout is considered a distinct state of psychological stress generated by the individual’s occupation and/or workplace and is identified as such in the World Health Organisation’s International Classification of Diseases [12, 13]. Recent research has focused on interventions to assist doctors in developing the skills and personal attributes needed to manage occupational stress and increase personal resiliency. The purpose of the present paper was to systematically review this literature, in order to critically evaluate and synthesise the available evidence for the effectiveness of psychosocial interventions in reducing burnout and stress among medical doctors. The results of this review are expected to provide doctors, hospital and organisational stakeholders, educators, and policy makers with a guide to the outcomes and current state of evidence for these programmes.

## Background

### Stress and burnout in medicine

Occupational stress occurs when job-related factors interact with individual factors, resulting in a change in the individual’s psychological and/or physiological state [14]. Burnout is a specific type of occupational stress and involves symptoms of emotional exhaustion, depersonalisation, and reduced feelings of personal accomplishment [15, 16]. It is a syndrome that is common among those working in the helping professions and is thought to be the result of the ongoing emotional demands associated with these occupations [4, 17].

The effects of burnout can be substantial, not only for doctors but also their patients. Burnout has been associated with significantly greater risk of making errors (e.g. medication errors, diagnostic and decision making errors) and suboptimal attitudes to patients (e.g. paying little attention to the social or personal impact of an illness) [2, 18]. Furthermore, burnout has been found to be an independent predictor of self-reported major medical errors [8], even after controlling for a range of personal and professional factors [6].

At an individual level, burnout among doctors has been associated with lower career satisfaction, higher absenteeism, greater probability of leaving the profession prematurely or choosing early retirement, and greater risk of experiencing difficulties in interpersonal relationships, such as with family and partners [3, 19, 20]. Many of these individual factors, such as absenteeism, job turnover, and early retirement, also result in adverse effects at the organisational level with burnout being associated with reduced workplace productivity and efficiency, reduced practice revenue, and greater probability of ordering unnecessary tests or procedures. Collectively, these factors result in greater unnecessary medical costs (direct and indirect) and patient burden [20]. It is clear that not only is burnout a widespread concern among the medical profession but also interventions to reduce stress and burnout are in the interests of doctors, organisations, and, most importantly, patients.

### Interventions to reduce occupational stress and burnout

Interventions to reduce occupational stress and burnout among doctors have primarily focused on changing organisational policies and procedures, such as reducing working hours, caseloads, and on call periods (e.g. [21–23]). The effects of such interventions focussing exclusively on organisational factors have been mixed (e.g. [24]), indicating that burnout is likely to be the result of both individual and organisational level processes. Further, the nature of medical practice (e.g. continuous exposure to situations that require doctors to provide medical care that can have life and death consequences) is not amenable to change, and thus, interventions directed at the organisational level are useful, but have restricted potential. Despite the large body of research examining prevalence, correlates, and effects of burnout among medical doctors, comparatively little research has focused on investigating psychosocial interventions to reduce occupational stress and burnout in this occupation. A systematic review conducted by McCray and colleagues [3] identified limited research on effective interventions for stress and burnout among doctors and a lack of quality and methodological rigour in the studies that had been conducted. In the nearly 10 years since this review was conducted, there has been increased attention and research in this field, with a greater number of controlled trials and large-scale cohort studies now available (e.g. [25–27]). As such, a systematic review of the literature to gauge the effects of these interventions and to guide the development of programmes, policy, and interventions is both timely and necessary.

### The current review

The aim of the current study was to systematically review evidence on psychosocial/behavioural interventions targeting stress and burnout among medical doctors. The review aimed to answer a number of questions, in particular regarding (1) the overall efficacy of interventions to reduce stress and burnout among doctors, (2) the relative efficacy of interventions by theoretical basis and type of intervention, and (3) the overall quality of research in the area. To address the first two aims, studies were assessed for the possibility of conducting pooled effect size analyses to aid data synthesis. Also of interest were the delivery format and duration of interventions, engagement strategies, populations investigated, and acceptability and satisfaction with interventions among doctors. Search and data extraction strategies were designed to target these key areas of interest.

## Methods

### Search strategy

A systematic review was conducted to identify articles published prior to January 2016, which investigated interventions for managing stress and burnout among medical doctors. This systematic review adhered to the Preferred Reporting Items for Systematic Reviews (PRISMA) checklist ([28], PRISMA checklist contained in Additional file 1), and a publically available protocol was registered prior to conducting the review (PROSPERO, registration number: CRD42016032595, http://www.crd.york.ac.uk/PROSPERO/). Five electronic databases, PsycINFO, Medline, Informit, CINAHL, and ProQuest Dissertations and Theses, were searched using database subject headings (e.g. MeSH terms) and text searches with key words (e.g. burnout, stress, physician, doctor). The specific search strategies for each database are outlined in Appendix 1. In addition, a manual search for relevant articles was also conducted using Google Scholar and ancestral searches through the reference lists of articles included in the final review. Searches were limited to studies written in the English language and original research papers (i.e. rather than conference proceedings, literature reviews, or summaries of interviews). From the initial search, titles and abstracts of articles were screened, followed by full-text screening. Searches, eligibility assessment, and data extraction were performed independently in an unblinded standardised manner by two reviewers. Discrepancies between reviewers were resolved by discussion and consensus with a third reviewer.

### Study selection

The review targeted quantitative intervention evaluations. For inclusion, studies were required to (1) be original research, (2) report on a psychosocial intervention targeting individual level stress or burnout, and (3) be tested among medical doctors as recipients of the intervention. As a minimum design required for inclusion, studies were required to report on the efficacy of an intervention using at least two time points, for example use of a pre-post design, rather than an intervention description or analysis of baseline characteristics of participants utilising a service. However, restrictions were not made regarding use of comparator conditions or random allocation, nor by field of specialisation or practice setting (e.g. hospital, community, private practice). Instead, the review focused broadly on summarising all available evidence in this emerging field.

Studies that did not directly assess occupational stress or burnout among doctors (e.g. depression, anxiety, or substance use) were excluded, as were studies that focused on organisational level interventions such as changes to policies, procedures, or management (e.g. changes to doctors’ working hours or on-call procedures), and studies that focused only on acceptability or satisfaction with an intervention without reporting intervention effects. Although studies were not restricted by level of training from registration as a doctor (e.g. intern, registrar, consultant), studies reporting on interventions for students were excluded.

### Data extraction

Criteria for data extraction were determined prior to review. The primary outcome measures were stress and burnout. No restrictions were placed on how these outcomes needed to be measured, for example whether by physiological or self-report means. Summary data for each study included design, participants, context/setting (e.g. hospital or community), stated primary and secondary outcomes, intervention details (theoretical underpinnings, duration, delivery format), outcomes at post and follow-up, and data relating to acceptability or participant satisfaction with the intervention. Extracted data were synthesised descriptively. Where possible, effect sizes and confidence intervals of effect sizes were extracted or estimated [29] to examine pooled effect sizes and risk of publication bias. Publication bias was intended to be assessed through examination of funnel plots. The quality of each study was evaluated according to the Critical Appraisal Skills Programme (CASP [30]) guidelines, with extracted data used to grade the level of evidence of each study according to the Strength of Recommendation Taxonomy (SORT [31]).

## Results

**Fig. 1:**
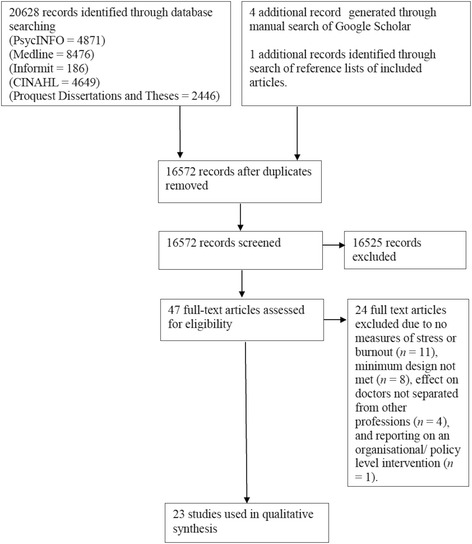
Flowchart showing results of the systematic review for studies investigating interventions to reduce occupational stress or burnout among medical doctors

Figure 1 displays the results of the systematic review article selection process. As our search strategy was purposefully broad and sensitive, the initial database search generated 20, 628 results with four additional articles identified through the Google Scholar search and one additional article identified through the ancestral search. After screening, 23 studies met criteria for inclusion in the review. Data summarising key methods and intervention effects are contained in Table 1.

**Table 1:** Summary of included intervention studies targeting stress or burnout among medical doctors

Study	Design	Participants, context/setting	Primary outcome(s)	Secondary outcome(s)	Intervention(s)	Outcome—post	Outcomes—follow-up	Acceptability/satisfaction	Limitations
Arora et al. [40]	RCT with participants allocated to intervention (*n* = 10) or active control (*n* = 10)	*N* = 18 (20 began) novice surgeons/UK	• Stress (STAI, heart rate, and salivary cortisol)	• Mental imagery	5 × 30 min mental practice sessions, each undertaken before performing a surgical procedure on a VR simulator	• Sig lower stress in intervention group than control on average and maximum heart rate and cortisol during intervention measurement points. Sig lower anxiety on STAI in intervention group. However, differences were not significant between the groups by the post measurement time point. • Sig higher mental imagery for intervention than control group	Not reported	Not reported	• ESs and CIs of ESs unable to be estimated • Unclear whether mental training would translate to stress reductions outside of the VR task • No intention to treat analysis conducted
Bar-Sela et al. [46]	Single group pre-post intervention design	*N* = 17 oncology residents/Israel	• Burnout (MBI)	• Expectations of contribution to improvement in abilities as doctors	9 × 1.5 h Balint group sessions, held monthly	• Means were divided by experience level (junior <3 years and senior >3 years). Across the three MBI scales, scores increased from beginning to end of year with the exception of depersonalisation among junior participants, which dropped. No statistical testing of changes	Not reported	• Sig increase from pre to post on residents’ ratings of contribution of intervention to abilities, although still only mid (3.1) on 5-point scale	• No control group • Insufficient sample size • No statistical testing of effects • ESs and CIs of ESs unable to be estimated
Bragard et al. [45]	RCT with participants allocated to intervention (*n* = 49) or waitlist control (*n* = 47)	*N* = 96 (of 2160 invited) medical residents working in oncology/Belgium	• Communication self-efficacy • Communication stress • Self efficacy to manage stress (all original questionnaires)	• Burnout (MBI)	40-h communication and stress management skills training programme, delivered in small groups (*n* = 7) over 8 weeks	• Relative to control group, intervention group reported sig less stress to communicate (*d* = .60, 95% CI = .21 to 1.04), sig greater self-efficacy to communicate (*d* = .88, 95% CI = .55 to 1.41) and self-efficacy to manage stress (*d* = .81, 95% CI = .45 to 1.3) • No sig differences on burnout subscales of EE (*d* = .12, 95% CI = −.29 to .52), DP (*d* = .02, 95% CI = −.27 to .54), or PA (*d* = .14, 95% CI = −.38 to .43)	Not reported	Not reported	• Original items used for stress to communicate, self-efficacy to communicate, and self- efficacy to manage stress showed poor test retest reliability (*r*s = .49, .51, and .58, respectively). Changes observed in scores should be interpreted with caution. • Average adherence to training programme was 50% • Low response rate • CIs of ESs estimated, not reported
Ghetti et al. [47]	Single group, pre-post intervention	*N* = 17 (of 36 invited) obstetrics and gynaecology residents/USA	• Burnout (MBI)	• Interest and ability in psychological medicine • Physician empathy	Balint groups, conducted once per month, each of 1-h duration, over 1-year intervention period	• No sig changes in burnout on subscales or total MBI • No sig changes in total interest and ability in psychological medicine, however at item level sig changes on 3 items (7, 8, and 9; relating to the doctor’s capacity to integrate psychological care into patient treatment) • No sig changes on physician empathy scale	Not reported	Not reported	• No comparison group • ESs and CIs of ESs unable to be estimated • Less than half of residents participating in the Balint groups completed measures • No data on average attendance in group • Potentially insufficient power
Gunasingam et al. [26]	RCT with participants allocated to intervention (*n* = 13) or waitlist control (*n* = 18)	*N =* 31 (of 52 invited) first year doctors based in a single metropolitan hospital/Australia	• Burnout (MBI)	• Acceptability and satisfaction with intervention (original questionnaire and qualitative focus groups)	Four debriefing sessions held over 8 weeks, each of 1-h duration, led by experienced senior health professional	• No sig changes in burnout on subscales or total MBI	Not reported	Well received—60% would recommend to future doctors and 90% found sessions to be a source of emotional and social support	• Weak statistical power • No data on attendance at sessions reported • ESs and CIs of ESs unable to be estimated • Passive control
Isaksson et al. [25]	Single group design, measures taken pre- and 1 year following intervention. Quasi comparison group to general sample of Norwegian doctors	*N* = 227 doctors (185 completed follow-up) self-referred for counselling between 2003 and 2005, compared with data from survey of 390 Norwegian doctors conducted in 2003/Norway	• Burnout (MBI)	• Mental distress • Job stress • Emotion focused coping and active coping • Neuroticism • Sick leave • Satisfaction with intervention	Doctors chose one of two interventions: a single day (6–7 h) individual counselling session or 5-day group-based counselling programme aimed at motivating reflection on the doctors’ situation and personal needs.	No post- intervention outcomes reported, only 12-month follow-up	(At 1 year) • Sig reductions in EE (*d* = .55, 95% CI = .33 to .77), DP (*d* = .28, 95% CI = .06 to .49), and PA (*d* = .08, 95% CI = −.13 to .29). • Sig reductions in mental distress, job stress (*d* = .65, 95% CI = .44 to .87), emotion focused coping and neuroticism • No sig change in active coping • Sig fewer doctors on full time sick leave	Descriptive statistics not reported, however among male doctors satisfaction with the intervention independently predicted reduction in EE	• ESs and CIs of ESs not reported, but estimated from presented data • No comparison condition • Poorly defined treatment • Effects of the two different counselling interventions not investigated separately • Some participants participated in psychotherapy after the programme, effect not accounted for in analyses • Reduction in work-hours confounds treatment effect and not controlled for
Isaksson et al. [50]	Single group design, measures taken 3 years following the intervention described in	*N* = 227 doctors (184 completed follow-up) self-referred for counselling between 2003 and 2005/Norway	• Burnout (MBI)	• Job stress • Emotion focused coping and active coping • Neuroticism • Sick leave	Doctors chose one of two interventions: a single day (6–7 h) individual counselling session or 5-day group-based counselling programme aimed at motivating reflection on the doctors’ situation and personal needs.	No post-intervention outcomes reported	(At 3 years) • No sig change in EE, job stress, emotion focused coping, and active coping from 1 to 3 years • Sig reduction in neuroticism from 1 to 3 years • From baseline (Isaksson et al. [25]) to 3-year follow-up, sig reductions in EE (*d* = .70, 95% CI = .48 to .93), and job stress (*d* = .77, 95% CI = .55 to .98)	Not reported	• ESs and CIs of ESs not reported, but estimated from presented data • No comparison condition • Poorly defined treatment • Effects of the two different counselling interventions not investigated separately • Reduction in work-hours confounds treatment effect and not controlled for
Kotb et al. [32]	Single group design, measures taken pre- and post- intervention	*N* = 33 family practice physicians/Egypt	• Burnout (MBI)	• Satisfaction with programme	Educational intervention consisting of 7 sessions, each of 60-min duration, focussing on CBT skills such as cognitive restructuring and relaxation	(At 6 months) • No sig changes in MBI subscales of EE (*d* < .01, 95% CI = −.75 to .74), DP (*d* = −.59, 95% CI = −1.37 to .18), or PA (*d* = −.08, 95% CI = −.82 to .67)	Not reported	81% of participants reported they were satisfied (score ≥60%) with the intervention	• ESs and CIs of ESs not reported, but estimated from presented data • No comparison condition
Krasner et al. [19]	Single group design, measures taken pre- (×2), post intensive period, post maintenance period, and 3 months following intervention	*N* = 70 (of 871 invited, 51 provided data at last assessment) primary care doctors in a continuing medical education course/USA	• Burnout (MBI)	• Mindfulness • Physician Empathy • Beliefs about psychosocial aspects of patient care • Personality • Mood states	Mindfulness, awareness, and communication training intervention, with 8-week intensive period (27 h total) and 10-month maintenance period (2.5-h session each month)	(Post maintenance) • Sig improvements on EE and DP, but not PA subscales of MBI • Sig improvements in total mood disturbance and physician beliefs • Sig increases in physician empathy, mindfulness, and Big 5 personality Mini-Markers	(At 3 months) • Sig reductions (from baseline) on EE (*d* = .62), DP (*d* = .45) and increase on PA (*d* = .44) subscales of MBI remained sig at follow-up • Changes observed on total scales for secondary outcomes from pre- to-post remained sig at follow-up	Not reported	• No comparison condition • Average attendance low (33.6 of possible 52 h) and not explored in relation to treatment phase or outcomes • ESs and CIs of ESs not able to be calculated for post time point
Lemaire et al. [37]	RCT with participants allocated to intervention (*n* = 21) or waitlist control (*n* = 19). Control group received intervention during follow-up period.	*N =* 40 hospital based physicians/Canada	• Stress (purposely constructed questionnaire using items from previously validated stress measures)	• Adherence • Heart rate • Blood pressure • Salivary cortisol	Biofeedback intervention delivered over 28 days, with 1 workshop (30 min), twice weekly meetings for intervention group, and practice 3 times per day for 5 min each.	• Sig reduction in stress (*d =* .44, 95% CI = −.19 to 1.07) for intervention group but not control group • No sig changes in blood pressure (systolic *d* < .01, 95% CI = −.62 to .62, diastolic *d =* .21, 95% CI = −.41 to .83), heart rate (*d* = .18, 95% CI = −.44 to .80), or salivary cortisol (*d* = .07, 95% CI = −.69 to .55) • 30% participants met criteria for “good” adherence	(At 4 weeks) • Sig reduction in stress for control group (after exposed to unsupported version of intervention) • Changes maintained for intervention group • No sig changes on physiological measures	Not reported	• Measure of stress not validated • Groups not equivalent on stress or heart rate at baseline • ESs and CIs of ESs not reported, but estimated from presented data
Maher et al. [42]	Blinded, matched, quasi-experimental design, with participants allocated to intervention (*n* = 11) or passive control (*n* = 15) conditions	*N =* 26 surgical residents/USA	• Stress (measure by STAI, heart rate, and subjective stress scale) • Surgical performance (blinded observer and self-assessed on OSATS)	• Acceptability of intervention	Stress management workshops (3 × 3 h duration, held weekly) focusing on identification of triggers and development of stress management techniques	• No significant effects on any measures, including between group differences on the STAI (*d* = .39) or heart rate variability (*d* = .56)	Not reported	• 91% of residents rated the intervention as valuable, 100% rated the intervention as good, very good, or excellent	• Non-random assignment of participants • Small sample/low statistical power • Use of ITT reported but final completion sample size not reported • Unclear if groups equivalent at baseline on DVs • ESs and CIs of ESs not reported, but estimated from presented data
Margalit et al. [36]	RCT with participants randomly allocated to didactic (*n* = 22) or interactive intervention (*n* = 22)	*N* = 44 (of 102 invited) general practitioners/Israel	• Knowledge • Management Intentions • Attitudes (including burnout assessed by a combination of items drawn from previously published measures of burnout)	N/A	Didactic or interactive (role play, Balint groups, individual teaching) instruction in bio-psychosocial approach to patient care. Workshops once per week for 4–6 h over 12-week period.	(At 6 months) • Sig increase in burnout (*d* = .46, 95% CI = .04 to .88) and burnout guilt feelings (*d* = .48, 95% CI = .06 to .90) for both groups • Sig increases in knowledge, intentions, patient centred attitudes, and self-esteem • Sig greater improvements on self-esteem and intentions for interactive learning group	Not reported	Not reported	• Adherence/participation not measured • Satisfaction not measured • Low opt in • Un-validated questionnaire • Poor internal consistency for work strain and burnout associated with guilt feelings subscales • CIs of ESs not reported, but estimated from presented data
McCue and Sachs [44]	Quasi-experimental trial, non-random allocation of doctors available to the intervention (*n* = 43) and doctors unavailable allocated to the control (*n* = 21)	*N =* 64 resident doctors in medicine, paediatrics, and medicine-paediatrics/USA	• Burnout (MBI) • Stress (ESSI)	• Life experiences (only completed by intervention participants)	Stress management workshop of 4-h duration emphasising personal management, relationship, outlook, and stamina skills	(At 8 weeks) • Sig reduction in stress • No sig changes on MBI subscales	Not reported	Evaluations indicated satisfaction with intervention, although no formal quantitative or qualitative analysis reported.	• Non-random allocation limits findings • ESs and CIs of ESs unable to be estimated • Pre-intervention equivalency of groups not established
Milstein et al. [43]	RCT with participants allocated to intervention (*n* = 7) or waitlist control (*n* = 8)	*N =* 15 (of 33 invited) paediatric house officers/USA	• Burnout (MBI)	• Utility, effectiveness, and deterrents of BATHE technique (qualitative interviews)	Instruction (45 min) in use of BATHE psychotherapeutic tool, focusing on awareness and self-empathy. Encouraged practise of tool 3 times per week for next 3 months.	(At 3 months) • No sig differences between groups or over time on MBI	Not reported	Not reported	• ESs and CIs of ESs unable to be estimated • Insufficient statistical power • Passive control condition • Minimum statistical detail not reported in results (no group *M*s or *SD*s)
Ospina-Kammerer and Figley [35]	Quasi-experimental trial, non-random allocation of doctors available to the intervention (*n* = 14) and doctors unavailable allocated to the control (*n* = 10)	*N* = 24 family practice residents/USA	• Burnout (EE subscale of MBI)	N/A	Relaxation/meditation training using Respiratory One Method (ROM). Participation in 4 workshops, held weekly, of 1-h duration.	• Sig lower EE in treatment group than control group at post treatment	Not reported	Not reported	• ESs and CIs of ESs unable to be estimated • Non-random allocation limits findings • Average attendance at workshops not reported • Small sample size
Pflugeisen et al. [39]	Single group pre-post intervention design (with 8-week follow-up)	*N* = 19 (of 23 originally enrolled) community hospital physicians/USA	• Burnout (MBI) • Stress (PSS)	• Mindfulness skills • Participant use of intervention aspects	Mindfulness intervention delivered via 3 live sessions (90 min each), 8 online training videos (5–7 min each), and weekly teleconference coaching calls (1 h each) delivered over 8 weeks.	• Sig increase in PA (*d* = .57, 95% CI = −.07 to 1.20) and reduction in stress (*d* = .88, 95% CI = .23 to 1.54). No sig changes for EE (*d* = .47, 95% CI = −.16 to 1.10) or DP (*d* = .33, 95% CI = −.30 to .95) • Sig increases in all mindfulness skills	(At 8 weeks) • Sig decrease in EE (*d* = .69, 95% CI = .05 to 1.33), stress (*d* = 1.14, 95% CI = .47 to 1.81), and increase in PA (*d* = .62, 95% CI = −.02 to 1.25) compared to baseline • Sig increases in all mindfulness skills except observing	Not reported	• Low participant adherence to reporting usage of intervention skills to researchers • Participation/attendance not reported • No control group • CIs of ESs not reported, but estimated from presented data
Popenoe [33]	Quasi- experimental trial, with participants allocated to burnout intervention (*n* = 3) or Balint group (*n* = 3) by pre-existing work groups	*N =* 6 family practice residents/USA	• Burnout (MBI) • Anxiety (STAI)	• Coping skills • Satisfaction with intervention	Four sessions (once a week, of 1-h duration) focusing on understanding burnout and developing coping skills to manage burnout (burnout group) or focusing on interactions with patients (Balint group)	• Burnout increased for all Balint group members and decreased for burnout group members, but no tests of significance conducted • No consistent differences between groups on anxiety or coping	Not reported	Stronger participant support for the value of the burnout group than for the Balint group	• No group level analyses of significance or individual level analyses of clinical change • ESs and CIs of ESs unable to be estimated • Small sample size • No baseline checks of equivalence • Non-random allocation limits findings
Shinefield [38]	Quasi-experimental trial, with non-random allocation of participants to intervention (*n* = 25) or waitlist control (*n* = 25)	*N* = 50 hospital based physicians/USA	• Burnout (MBI)	• Personality • Coping strategies • Intervention satisfaction	Six-week stress reduction training programme based on cognitive behavioural principles, with sessions held weekly for 2-h duration	• The treatment group reported sig improvement on burnout (MBI subscales of EE *d* = 1.06, 95% CI = .64 to 1.49, DP *d =* .59, 95% CI = .18 to 1.00, and PA *d =* .30, 95% CI = −.11 to .70) relative to the control group • No sig interactions between personality type and intervention effects	Not reported	Overall satisfaction was high. Components most preferred by participants were relaxation and assertion, self-care, and exercise.	• Non-random allocation limits findings • ESs and CIs of ESs not reported, but estimated from presented data • Baseline differences between groups on number of patients seen per week, burnout, and job satisfaction not controlled. • Passive control condition
Sood et al. [48]	RCT with participants allocated to intervention (*n* = 20) or waitlist control (*n* = 20)	*N =* 32 (of 40 enrolled) internal medicine physicians/USA	• Stress (PSS) • Resilience (CD-RISC)	• Anxiety • Fatigue • Quality of life	Single, 90-min individual training in SMART resiliency and stress management programme, and training in a paced breathing meditation	(At 8 weeks) • Sig increase in resilience (*d* = 1.16, 95% CI = .38 to 1.98), decrease in stress (*d* = 1.01, 95% CI = .22 to 1.79), decrease in anxiety (*d* = 1.32, 95% CI = .51 to 2.15), and increase in quality of life (*d* = .83, 95% CI = .06 to 1.61) for intervention group compared to control. • No sig change for fatigue (*d* = .23, 95% CI = −.48 to 1.02)	Not reported	Not reported	• Differential attrition between control and intervention groups • Passive control condition • CIs of ESs not reported, but estimated from presented data
Sood et al. [27]	RCT with participants allocated to intervention (*n* = 13) or waitlist control (*n* = 13)	*N =* 26 radiology physicians/USA	• Stress (PSS) • Resilience (CD-RISC)	• Anxiety • Mindful awareness • Quality of life	Single, 90-min individual training in SMART resiliency and stress management programme, with two follow-up phone calls and optional 30-min booster session	(At 12 weeks) • Sig decreases in stress (*d* = .98, 95% CI = .12 to 1.83), anxiety (*d* = .86, 95% CI = .02 to 1.71), and increases in mindful attention awareness (*d* = 1.24, 95% CI = .36 to 2.13), and quality of life (*d* = .83, 95% CI = −.01 to 1.68) for intervention group compared to control • No sig difference for resilience (*d* = .35, 95% CI = −.47 to 1.16)	Not reported	Not reported	• Incomplete data regarding attrition • Apriori sample size not reached • Passive control group • CIs of ESs not reported, but estimated from presented data
West et al. [49]	RCT with participants allocated to intervention (*n* = 37) or active control (*n* = 37)	*N =* 74 internal medicine physicians/USA	• Burnout (MBI) • Stress (PSS)	• Job satisfaction • Quality of life • Fatigue • Physical and mental health • Depression • Physician empathy	Guided group discussions (1-h duration, held fortnightly over 9 months, 19 sessions in total) focussing on mindfulness, reflection, and shared experiences	• No sig differences on for total burnout (*d* = .03, 95% CI = -.44 to .50) or stress (*d* = .02, 95% CI = -.44 to .50)	(At 3 months) • Sig increased engagement and reduced high item DP (on MBI) for intervention group compared to control *d* = .70, 95% CI = .23 to 1.19, with effects maintained at 12-month follow-up	Not reported	• Intervention attendance low (average of 11.7 of 19 sessions attended)
Wetzel et al. [41]	RCT with participants allocated to intervention (*n* = 8) or waitlist control (*n* = 8)	*N =* 16 surgical residents/UK	• Stress (heart rate, salivary cortisol, and observer rating) • Anxiety (STAI)	• Applied surgical coping strategies • Acceptability of intervention	Stress management training including relaxation, coping, and mental rehearsal strategies, duration not provided	• Sig lower coefficient of heart rate variability (*d* = 1.70, 95% CI = .55 to 2.84), higher observational team work in surgery (*d* = .71, 95% CI = −.30 to 1.72) & greater coping skills in intervention • No sig differences on STAI (*d* = .18, 95% CI = .80 to 1.16) or cortisol (*d* = −.36, 95% CI = −1.35 to .63)	Not reported	Qualitative feedback indicated perceptions of improved skills and confidence as a result of the intervention	• ESs and CIs of ESs not reported, but estimated from presented data • Passive control group • Insufficient statistical power
Winefield et al. [34]	Single group design, measures taken pre- and post-intervention	*N =* 20 female general practitioners/Australia	• Burnout (MBI) • Psychological distress (GHQ-12) • Job satisfaction	• Job satisfaction • Satisfaction with intervention	Three educational seminars, held fortnightly, each of 3-h duration, focusing on relaxation training, social support, managing self-expectations, and practice management	(At 4 weeks post intervention) • Sig reductions in EE subscale of MBI (*d* = .37, 95% CI = −.24 to .98) and psychological distress (*d* = 1.13, 95% CI = .49 to 1.80) • No sig differences for DP (*d* = .19, 95% CI = −.43 to .79) or PA (*d* = .25, 95% CI = −.36 to .86) subscales of MBI, or job satisfaction	Not reported	High satisfaction with programme, with group discussions rated as most helpful activity	• No comparison group • ESs and CIs of ESs not reported, but estimated from presented data • Some evidence of regression to the mean

Sample effect sizes are reported wherever it was possible to calculate them; however, it should be noted that for non-significant effects, the population effect size cannot be reliably inferred to be anything but zero*. RCT* randomised controlled trial, *ES* effect size, *CI* confidence interval, *STAI* State-Trait Anxiety Inventory, *MBI* Maslach Burnout Inventory, *EE* emotional exhaustion subscale of MBI, *DP* depersonalisation subscale of MBI, *PA* personal accomplishment subscale of MBI, *ESSI* Stress Systems Instrument, *PSS* Perceived Stress Scale, *CD-RISC* Connor-Davidson Resilience Scale, *GHQ-12* General Health Questionnaire, *OSATS* Objective Structured Assessment of Technical Skill, *ITT* intention to treat, *DVs* dependent variables

### Participants, contexts, and design

The 23 studies were conducted with participants across a range of medical specialties, with family or primary care doctors being the most commonly studied population [19, 32–36]. Studies were also conducted among general hospital-based [26, 37–39], surgical [40–42], paediatric [43, 44], oncology [45, 46], obstetrics and gynaecology [47], internal medicine [48, 49], and radiology [27] fields of practice. Two studies utilised samples of doctors from a range of specialties and work settings who were accessing the intervention service through a nationally available programme [25, 50]. The majority (12 of the 23 studies) of research was conducted in the USA, with only one study [32] conducted in a developing nation. The studies were typically underpowered or of small samples size, ranging from 6 [33] to 227 participants [25, 50], with only eight studies reporting samples greater than 40 participants [19, 25, 36, 38, 44, 45, 49, 50]. For the 15 studies with multiple conditions, the average condition size was 17.67 (SD = 11.77). The majority (67%, *n* = 10) of these studies had less than 20 participants per condition, which limits any interpretations of population efficacy [51].

Eight studies were conducted as single group pre-post intervention designs with no comparison conditions [19, 25, 32, 34, 39, 46, 47, 50] and 10 were conducted as randomised controlled trials (RCTs) [26, 27, 36, 37, 40, 41, 43, 45, 48, 49]. A further five studies [33, 35, 38, 42, 44] utilised quasi-experimental designs with non-random allocation of participants to groups, which was typically based on doctor availability to participate in intervention activities.

Of the 15 studies that included two or more experimental arms (RCTs and quasi-experimental designs), only four [33, 36, 40, 49] utilised active control conditions. The remaining studies utilised passive waitlist control comparators. Such designs are problematic, particularly when examining occupational stress or burnout, as the time allocated away from regular duties to undertake intervention activities may itself facilitate change rather than any specific intervention strategy. This problem may be further compounded in those studies that allowed self-selection of participants to interventions based on work availability. That is, the doctors electing the control groups may have already been under greater work strain (e.g. [38]) and were then not given the equivalent time released from work as those participants undertaking the intervention, thus only creating the illusion of specific intervention effects. Furthermore, self-selection also creates the possibility that participants may have selected conditions based on other variables, such as personality traits, which may then also have influenced engagement with, and the results of, interventions. Of the four studies that contained active comparison conditions, two [40, 49] utilised active control conditions, i.e. participants in the control condition were released from regular duties for the same period of time or undertook an unrelated task for the period, and two studies compared two comparable active treatment interventions [33, 36].

### Delivery format, duration, and engagement

All interventions were delivered in person, although detail was often lacking regarding the skills and training of the individuals delivering the interventions. Total duration of interventions varied from 45 min [43] to approximately 60 h [36], with one study failing to specify the duration of the intervention [41]. Most studies (12 out of 13) were brief interventions of less than 10-h duration [26, 27, 32–35, 37, 40, 42–44, 48]. The two studies conducted by Isaksson and colleagues [25, 50] combined participants who completed a 1-day (6–7 h) counselling and participants who completed a 5-day counselling intervention, although specific intervention effects for the different programmes were not explored. No clear dose-response patterns were identified among interventions, with strong treatment effects reported for the brief intervention examined by Sood et al. [27, 48], but increases in burnout found for the 60-h intervention examined by Margalit et al. [36]. There was a lack of detail in describing and utilising strategies to promote engagement with interventions, which may have contributed to the low levels of participant adherence observed in a number of the studies (e.g. [19, 45, 49]). Detail was also lacking in measuring and reporting engagement and adherence with intervention procedures. From the 23 studies, only 43% (*n* = 10) reported data relating to participant adherence or participation in intervention procedures and 74% (*n* = 17) reported data relating to participant dropout during the intervention.

### Research quality

Studies were appraised for quality in accordance with the CASP and SORT guidelines (see Table 2). Results of these analyses indicated that there is a need for improved quality among studies conducted in this area. In particular, many studies lacked detail in reporting of statistical analyses and/or failed to adequately check for and control baseline differences between groups. There was insufficient use of random allocation of participants and active control conditions. Insufficient reporting on participant flow made it difficult to determine the level of dropout in studies, and there were only limited attempts to account/adjust for the effect of this dropout on main analyses, such as by means of intention to treat. From the data extracted using the CASP, only nine studies (39% [19, 25, 27, 38, 39, 41, 45, 48, 50]) provided enough evidence to determine that the benefits of the intervention outweighed the costs or harms. Many studies did not provide adequate detail on effect sizes or main analyses for this decision to be made. The data extracted from the CASP and study summaries were used to rate the quality of each study according to the SORT. All 23 studies were rated as Level 2 Evidence in terms of quality, with no studies meeting criteria to be classified as high quality, Level 1 Evidence. Due to the inclusion criteria utilised pertaining to study design (measurements at least two time points), no studies were classified as Level of Evidence 3. The overall “Strength of Recommendation” for the body of evidence was classified as B, as consistent findings from at least two high quality (Level of Evidence 1) studies were not found.

**Table 2:** Summary of study quality based on the CASP randomised controlled trial checklist and the SORT

	CASP											SORT
Study	1. Did the study address a clearly focused issue?	2. Was the assignment of patients to treatments randomised?	3. Were patients, health workers, and study personnel blinded?	4. Were the groups similar at the start of the trial?	5. Aside from the experimental intervention, were the groups treated equally?	6. Were all of the patients who entered the trial properly accounted for at its conclusion?	7. How large was the treatment effect?^a^	8. How precise was the estimate of the treatment effect?^b^	9. Can the results be applied to other populations?	10. Were all clinically important outcomes considered?	11. Are the benefits worth the harms and the costs?^c^	Level of Evidence
Arora et al. [40]	Yes	Yes	Patients only	Yes	Yes	Yes	Insufficient results presented to determine	Not reported	Yes	Acceptability and satisfaction not considered	Uncertain	2
Bar-Sela et al. [46]	Yes	N/A (single sample)	N/A	N/A	N/A	Yes	Insufficient results presented to determine	Not reported	Yes	Participation/adherence not reported	Uncertain	2
Bragard et al. [45]	Yes	Yes	No	No	No (passive control condition)	No (11 were excluded for not completing all assessments, but no details were given)	Medium	Not reported	Yes	Acceptability and satisfaction not considered	Yes	2
Ghetti et al. [47]	Yes	N/A (single sample)	N/A	N/A	N/A	No	Insufficient results presented to determine	Not reported	No (less than half of participants in groups completed measures)	Acceptability, satisfaction, and participation/attendance not considered	Uncertain	2
Gunasingam et al. [26]	Yes	Yes	No	No	No (passive control condition)	Yes	Insufficient results presented to determine	Not reported	Yes	Doctor participation/attendance not considered	Uncertain	2
Isaksson et al. [25]	Yes	N/A (single sample)	N/A	N/A	N/A	Yes	Medium	Not reported	Yes	Yes	Yes	2
Isaksson et al. [50]	Yes	N/A (single sample)	N/A	N/A	N/A	No	Medium	Not reported	Yes	No (excluded 2 of 3 MBI scales as well as the symptom checklist that was previously administered at 12 month follow up	Yes	2
Kotb et al. [32]	Yes	N/A (single sample)	N/A	N/A	N/A	No	Small	Not reported	Yes	Yes	No	2
Krasner et al. [19]	Yes	N/A (single sample)	N/A	N/A	N/A	No	Medium (at follow up)	Not reported	Yes	Acceptability and satisfaction not considered	Yes	2
Lemaire et al. [37]	Yes	Yes	No	No	Yes	Yes	Small	Not reported	Yes	Acceptability and satisfaction not considered	No	2
Maher et al. [42]	Yes	No	Evaluators and researchers blinded, participants not	Unclear	No (passive control group)	Uncertain	Insufficient results presented to determine	Not reported	Yes	Yes	No	2
Margalit et al. [36]	Yes	Yes	No	Yes	Yes	Uncertain	Small negative effect	Not reported	Yes	Acceptability and satisfaction not considered	No	2
McCue and Sachs [44]	Yes	No	No	No	No (control group could not be released from work)	Yes	Insufficient results presented to determine	Not reported	Yes	Yes	Uncertain	2
Milstein et al. [43]	Yes	Yes	No	Yes	No (passive control condition)	Yes	Insufficient results presented to determine	Not reported	Uncertain (small sample size)	Acceptability and satisfaction not considered	Uncertain	2
Ospina-Kammerer and Figley [35]	Yes	No	No	Yes	Yes	Yes	Insufficient results presented to determine	Not reported	Yes	Total burnout scale not reported. No investigation of acceptability, satisfaction, or participation.	Uncertain	2
Pflugeisen et al. [39]	Yes	N/A (single sample)	N/A	N/A	N/A	No	Large for stress, medium for PA subscale	Not reported	Yes	Acceptability, satisfaction, and participation/adherence not considered	Yes	2
Popenoe [33]	Yes	No	No	Uncertain (no pre statistics reported)	Yes	Yes	Insufficient results presented to determine	Not reported	No	No (only the EE aspect of burnout considered)	Uncertain	2
Shinefield [38]	Yes	No	No	No	No	Yes	Medium	Not reported	Yes	Yes	Yes	2
Sood et al. [48]	Yes	Yes	No	Yes	No (passive control condition)	No	Large	Not reported	Yes	Acceptability and satisfaction not considered	Yes	2
Sood et al. [27]	Yes	Yes	No	Yes	No (passive control condition)	No	Large	Not reported	Yes	Acceptability and satisfaction not considered	Yes	2
West et al. [49]	Yes	Yes	No	Yes	Yes	Yes	Insufficient results presented to determine	Not reported	Yes	Acceptability and satisfaction not considered	Uncertain	2
Wetzel et al. [41]	Yes	Yes	No	No	No (passive control condition)	Yes	Large	Not reported	Yes	Yes	Yes	2
Winefield et al. [34]	Yes	N/A (single sample)	N/A	N/A	N/A	Yes	Small	Not reported	Yes	Yes	No	2

*MBI* Maslach Burnout Inventory, *EE* emotional exhaustion, *PA* personal accomplishment

^a^Based only on effect sizes for primary outcome (stress or burnout) measures

^b^Based on confidence intervals around an effect size

^c^For studies in which an effect size was not reported and not able to be calculated from the information provided, the weight of the benefits of the intervention were deemed unclear, and as such the item was entered as “uncertain”

### Interventions and effects

The 23 studies reported on 21 unique intervention programmes, with one nationally available counselling intervention reported in two studies [25, 50] and one resiliency and stress management programme also reported in two studies [27, 48]. The efficacy of interventions was examined with regard to theoretical basis or approach of the intervention. To achieve this, studies were grouped thematically in accordance with the primary features or targeted processes of change of the intervention. Through this process, three broad categories of interventions were identified; interventions that focused on educating or achieving cognitive or behavioural change, interventions that focused purely on relaxation or attention training strategies, and interventions that were primarily designed as unstructured support or discussion-focused.

#### Effect sizes and pooling

For studies that did not report effect sizes (*n* = 20, approximately 87%), these were estimated from descriptive or test statistics when such information was available [29]. Effect sizes were able to be calculated for 12 studies, but should be interpreted cautiously as they could not be adjusted for the correlation between pre-post assessments, as it was often not reported in the original study. Furthermore, three of these studies enabled calculation of effect sizes for a single subscale of burnout, but not for the remaining subscales or total scale. As total effect sizes were available for less than half of the study sample, a risk of bias analysis would not have been representative of the sample and may have been misleading and, therefore, is not reported.

Although pooling of effect sizes was intended as a method to assist in the synthesis of data from the study pool, the primary data could not support such an analysis and so a decision was made to proceed with a narrative only systematic review. This decision was made based on the following: the lack of available effect sizes or primary data to estimate effect sizes (half of total studies, none available from the category of discussion/support groups, and only one effect size from the relaxation category); lack of quality among the study sample; inclusion of randomised and non-randomised studies in the review; and heterogeneity among the included studies. Furthermore, with no studies being classified as high quality, meta-analysis was considered inappropriate as pooling effect sizes across studies where some or all are at elevated risk of internal bias may compound the errors of the original studies and produce incorrect and misleading results [52–54]. In addition, pooling effect sizes from both randomised and non-randomised studies presents a number of methodological concerns, which limits inferences and generalisability of meta-analytic claims [55]. Finally, among the one intervention category (cognitive behavioural interventions) that contained greater than one effect size (to allow for pooling), considerable heterogeneity was observed among the studies. This heterogeneity related to study design (one or multiple samples, random or non-random allocation, intervention and follow-up lengths), outcome measures (physiological or self-report, stress or burnout), and participant populations (junior doctor, specialist, or general samples). For these reasons, pooling of effect sizes was considered premature given the state of the current body of evidence.

#### Interventions based on cognitive or behavioural principles

Although there was considerable diversity in the interventions described within this group, 17 of 23 studies described interventions that were primarily based on principles of cognitive or behavioural change. These interventions aimed to promote the development of coping, stress management, mindfulness, communication, or cognitive reappraisal skills [19, 25, 27, 33, 34, 36, 38, 39, 41–45, 48–50, 51]. Of the 17 studies, six examined both stress and burnout [25, 39, 44, 45, 49, 50], seven examined only burnout [19, 33, 34, 36, 38, 43, 32], and four examined only stress [27, 41, 42, 48].

Among the 10 studies reporting on measures of stress, stress was measured by a variety of means, including self-report questionnaires, heart rate variability, and cortisol levels. Seven studies reported (or contained information required to estimate) at least one pre- to post-intervention effect size for stress, resulting in nine available effect sizes. Primarily positive, medium [45] to large [27, 39, 41, 48] reductions in stress were reported (effect sizes ranging *d =* .02–1.70) from pre- to post-intervention periods. Despite reporting a large reduction in coefficient of heart rate variability (*d* = 1.70), Wetzel and colleagues [41] reported only a small effect on a simultaneous cortisol measurement of stress (*d* = .36). One study reported a non-significant, but medium (*d =* .56) increase in mean heart rate variability for intervention participants compared to control participants [42]. An effect size was not able to be calculated for McCue and Sachs [44], although a significant reduction in stress was reported. Isaksson and colleagues reported outcomes of a counselling intervention across two papers, and although no post-treatment data was presented, a moderate reduction in job-related stress (*d =* .65) was reported at 12-month follow-up [25] and maintained at 3-year follow-up [50]. West et al. [49] reported no significant treatment effects for stress (*d* = .02) between intervention or active control conditions at post or either 3-month or 1-year follow up time points. Only one other paper examined follow up intervention effects for stress, with Pflugeisen et al. [39] reporting maintenance of treatment gains at an eight week follow-up.

Of the 13 cognitive/behavioural intervention studies that contained a measure of burnout (all utilising self-report assessments), seven contained (or allowed estimation of) at least one pre- to post-intervention effect size, with 12 effect sizes available across the articles. All except one study operationalised burnout according to the three subscales described by Maslach [16], with primarily small to medium (*d =* .08 to 1.06) reductions in burnout reported [32, 34, 38, 39, 49]. One study [32] reported gains on one burnout subscale (from the Maslach inventory), but no effect on other subscales from the same inventory (*d* < .01 to .08), and one further study reported no intervention effects (*d* = .02 to .14) on any burnout subscale [45]. Of the six studies, two measured burnout as a total scale, with [36] reporting a significant, but small (*d* = .46) increase in burnout from pre- to post-intervention, and [49] reporting non-significant total effects. Three studies did not contain post-intervention data, but did report on follow-up data. In the two articles by Isaksson and colleagues [25, 50], a medium reduction (*d* = .55) only on the emotional exhaustion subscale of the Maslach Burnout Inventory (MBI) was observed at 12-month follow-up and maintained at 3-year follow-up. Krasner et al. [19] also reported small to medium (*d* = .44 to .62) reductions across burnout subscales at their 3-month follow-up. Pflugeisen et al. [39] and West et al. [49] were the only studies to report both post and follow-up data, with Pflugeisen et al. [39] reporting treatment gains on the personal accomplishment and emotional exhaustion subscales of the MBI maintained at 8-week follow-up. West et al. [49] reported no overall or subscale changes at post, but a significant improvement in scores identified as high on the depersonalisation subscale at three-mont follow-up, which were maintained through to one-year follow-up. Of the three studies that did not report sufficient data to estimate effect size, one reported no tests of significance [33] and the remaining two [43, 44] reported non-significant changes in burnout from pre- to post-intervention.

#### Relaxation and attention training interventions

While a number of interventions using cognitive or behavioural strategies included relaxation or attention training as a component, three studies described interventions that focused solely on the use of relaxation or attention training to reduce occupational stress or burnout [35, 37, 40]. Arora et al. [40] utilised a mental imagery intervention designed to lower surgeons’ stress while performing a surgical procedure; Ospina-Kammerer and Figley [35] utilised a relaxation intervention focused on breathing to reduce burnout; and Lemaire et al. [37] utilised a biofeedback intervention based on participants’ heart rhythm patterns to reduce stress. Of the three studies, only Lemaire et al. [37] reported effect sizes, with a small (*d* = .44) reduction in self-reported stress observed at post-treatment and maintained at 4-week follow-up. This effect was not however replicated on their physiological measures of stress (*d* < .01 to .21). The remaining two studies did not allow for effect size estimation. However, Ospina-Kammerer and Figley [35] reported a significant reduction in the emotional exhaustion subscale of the MBI (results for other subscales not reported) relative to the control group at post treatment, and Arora et al. [40] reported a reduction in average and maximum heart rate and salivary cortisol during the intervention but not at post intervention. Neither study reported follow-up effects.

#### Discussion and support interventions

Three studies focused on the efficacy of support or discussion interventions [26, 46, 47], while a fourth utilised a discussion group as the comparator in examining the efficacy of their cognitive behavioural intervention [33]. Bar-Sela et al. [46], Ghetti et al. [47], and Popenoe [33] reported on the use of Balint groups, while Gunasingam et al. [26] reported on the use of workplace debriefing sessions. Effect sizes were not reported or able to be estimated for any of the studies. None of the studies reported significant intervention effects on measures of burnout, with Popenoe [33] and Bar-Sela et al. [46] reporting a trend for burnout scores to worsen over time for participants in the Balint group. These results should however be interpreted with caution, due to the small sample sizes and lack of individual or group level analyses, such as clinical change or analyses of significance or effect size.

### Acceptability and satisfaction

Studies were reviewed for assessment of participant acceptability or satisfaction with interventions, regardless of whether this was examined by quantitative or qualitative methods. Acceptability or satisfaction with the intervention was formally assessed in only 10 studies (44%). Among these, acceptability and satisfaction was typically assessed by means of original questionnaires or interviews, with data analysed descriptively, for example by mean ratings of satisfaction or percentage of satisfied participants. The use of original and unstandardised questionnaires limits comparisons and data synthesis across studies. However, for the studies that did report on these factors, acceptability and satisfaction were typically high, although this should be considered in the context of the use of unstandardised measures and that many lacked an active comparison condition against which to assess satisfaction. Isaksson [25] found that among male doctors’ satisfaction with the intervention independently predicted reduction in the emotional exhaustion scale of the MBI. However, this trend was not significant for female doctors. Although most studies did not formally assess satisfaction or acceptability, issues such as low opt in rates (e.g. [45, 47]) and low adherence to intervention procedures (e.g. [19, 45]) should be considered in the context of assessing acceptability and satisfaction. Overall, there is a need for greater use of standardised measures to assess intervention satisfaction (e.g. [56]) and greater rigour reporting and assessing participant adherence.

## Discussion

The principal aim of the current review was to evaluate and summarise evidence for the efficacy of psychosocial/behavioural interventions, targeting stress and burnout among medical doctors. Secondary aims were to identify whether the relative efficacy of these interventions varied according to theoretical basis or type of intervention and to also establish the overall quality of research in the area. An examination of these issues is necessary to determine whether occupational stress and burnout in medical doctors can be mitigated via such interventions and to provide a guide to the nature of the programme effects.

Of the 23 articles reviewed, approximately half the studies (*n* = 11), pre- to post-intervention effect sizes were not reported or insufficient data was reported to allow effects to be estimated, which limited capacity for a representative assessment of publication bias. Compounding this issue, a lack of quality among the reviewed studies, inclusion of randomised and non-randomised studies, and considerable heterogeneity among studies precluded the pooling of effect sizes as to do so with research of this type would have been inappropriate and potentially misleading [52–55]. This decision also prevented the statistical comparison of interventions across theoretical orientations (particularly with no discussion/support interventions reporting effect sizes and only one relaxation focused intervention reporting an effect size) or determining overall effects of psychosocial interventions for stress or burnout among medical doctors.

Within the CBT approaches, the strongest effects were reported for stress as an outcome and generally only moderate effects noted for burnout. Although interpretation of the effect sizes should be made with caution given the considerable proportion of studies that did not provide enough data to determine the magnitude of the effect, these results may suggest that the components of the CBT interventions studied here may not have adequately addressed burnout. Greater investigation of active treatment components to target burnout specifically is an important avenue for future research. The review also indicates that the efficacy of relaxation interventions may be promising, though this is based almost exclusively on statistical significance results and should be interpreted with caution due to the small number of studies (*n* = 3) and treatment effect sizes available (*n* = 1). No evidence, whether by effect size or statistical significance, was found for the efficacy of support of discussion groups, although again this is limited by the small sample size (*n* = 4). These conclusions are provisional and subject to change as further, high quality, evidence becomes available.

Given that burnout represents a specific type of occupational stress and incorporates potentially more intense and longer-term symptoms such as emotional exhaustion, depersonalisation, and reduced feelings of personal accomplishment, it is likely that more focused intervention strategies are required. The fact that support-based interventions failed to demonstrate benefit suggests that new learning is required with respect to coping or management strategies. That is, interventions may need to focus on facilitating the development of individually meaningful strategies for managing occupational stress in the longer term, to assist medical doctors in coping with work that is, by its nature, inherently challenging.

While this review highlights the potential of psychosocial interventions to reduce the negative impacts of occupational stress and burnout in medical doctors, caution is required in the interpretation of the findings. Although there has been increased attention and research in this field since the review conducted by McCray and colleagues [3], as indicated by the current quality appraisal, studies generally remain of moderate quality. McCray et al. [3] identified a need for improved quality and rigour within the field. In the nearly 10 years since this review, more than double the number of studies have been reviewed in the present paper, yet similar to the original review, no studies received the SORT 1 quality rating. Therefore, while research in this field has expanded, issues with quality persist despite calls for improvement. Quality appraisals in the present review identified a pressing need for well-powered, rigorous RCTs. Studies reviewed were often underpowered and lacking appropriate comparison groups, relevant statistical analysis, comprehensive assessment of treatment effects (group and individual level), long-term follow-up, and acceptability/feasibility data. Across studies, there was a need for greater consistency in reporting treatment outcomes (effect sizes of raw data that allows for further pooling of data), particularly given that many studies were lacking in statistical power. Thus, while psychosocial interventions may offer promise, recommendations regarding their use cannot yet be made with confidence.

Doctors’ acceptability and satisfaction with the programmes were generally high, but were directly assessed in only 10 of the 23 studies reviewed. Overall, there is a need for greater use of standardised measures to assess intervention satisfaction (e.g. [56]) and high quality qualitative and survey research to better understand the perceived needs of this population as well as the relative appeal of different intervention modalities. Combined with standardised approaches for assessing efficacy, this research would ensure that programmes meet the needs and expectations of doctors and thus have a greater chance of uptake. Greater rigour is also needed in reporting rates of participant adherence and dropout. While potential problems of adherence with psychosocial interventions are not necessarily unique to programmes targeting medical doctors, the focus for these interventions should be to reduce occupational stress while not adding burden to an individual’s workload. It is therefore vital to ensure such interventions are integrated into the workplace or the doctor’s lifestyle in a non-intrusive manner. Such strategies will be essential for efficacious programmes to reach optimal potential in terms of implementation and dissemination.

### Strengths and limitations

This review was conducted according to PRISMA guidelines and utilised established measures of quality assessment (CASP and SORT) in evaluating studies and the body of evidence. Despite these strengths, results of the review should also be considered within the context of a number of limitations. In particular, publication bias was not assessed (available data may not have been representative of the sample) and pooling of effect sizes was considered premature. Although these exclusions may be considered limitations of the review, it is the authors’ opinion that a narrative systematic review is the most appropriate approach for the current state of evidence in this field. This is also a key finding of the review itself and provides a clear indication of avenues for future research. However, results should be interpreted cautiously due to these exclusions. Furthermore, not discussed in this review was the cost of each intervention, financial or otherwise. This outcome was not included in the review due to a lack of reported information in the included studies. Cost-effectiveness is an important consideration of any intervention, and within this field, it may be of particular importance when viewed in the context of the costs that arise from the consequences (medical errors, staff absences, early retirement from the profession) of a population experiencing high stress and/or burnout. Lastly, considerable heterogeneity was found in the measurement of acceptability and satisfaction across studies. The present review has reported this data by individual study; however, it may be beneficial in future reviews to synthesise this data according to theme or facet of acceptability or satisfaction, for example, satisfaction with rationale, timing, or effects.

## Conclusion

Burnout is not only highly prevalent among the medical profession [1, 2] but also associated with significant costs to doctors, patients, and healthcare systems [2, 5–8]. This review has found that despite increased attention, the quality of research examining the benefits of psychosocial/behavioural interventions for occupational stress and burnout in medical doctors remains less than optimal. Despite this, interventions focused on cognitive and behavioural principles currently have the greatest evidence base and, to date, show promise as an efficacious treatment approach, particularly in reducing stress among doctors. There is also some support for the conclusions that this approach is moderately effective with respect to burnout.

This review highlights a pressing need for more research to be conducted, particularly high-quality RCTs, which will enable recommendations to be made about the relative efficacy of various psychosocial interventions, their ability to improve both stress and burnout, as well as produce long-term benefits and observable occupational improvements (e.g. associated improvements in medical errors, career satisfaction). Such research should also take into consideration the cost-effectiveness of available interventions, particularly with reference to the costs of not intervening. The challenge for hospital stakeholders, educators, and policy makers is to identify programmes that are effective for improving multiple outcomes, are acceptable, and can be easily integrated into training or practice to facilitate engagement with and uptake of the intervention.

## Appendix 1

### Database search strategies

#### Search strategy for PsycINFO

1. (intern OR interns OR internship OR resident OR residents OR physician OR “medic* practi*” OR doctor OR surgeon OR registrar).ab
2. (burnout OR stress OR resilien* OR fatigue).ab
3. Physicians/OR family physicians/OR general practitioners/OR gynaecologists/OR internists/OR neurologists/OR obstetricians/OR pathologists/OR paediatricians/OR psychiatrists/OR surgeons/
4. Occupational stress/OR stress/
5. 2 OR 4
6. 1 OR 3
7. 5 and 6
8. Limit 7 to English language

#### Search strategy for Medline

1. (intern OR interns OR internship OR resident OR residents OR physician OR “medic* practi*” OR doctor OR surgeon OR registrar).ab
2. (burnout OR stress OR resilien* OR fatigue).ab
3. physicians/OR foreign medical graduates/ OR general practitioners/ OR osteopathic physicians/ OR physicians, family/ OR physicians, primary care OR physicians, women/ OR surgeons/ occupational health physicians
4. burnout, professional/
5. 1 OR 3
6. 2 OR 4
7. 7 AND 6
8. limit 7 to English language

#### Search strategy for Informit

Databases selected for inclusion were: Australasian Medical Index (AMI), Australian Public Affairs Information Service - Health (APAIS-Health), Health & Society, Health Collection, Rural and Remote Health Database (RURAL).

1. (intern OR interns OR internship OR resident OR residents OR physician OR “medic* practi*” OR doctor OR surgeon OR registrar).ab
2. (burnout OR stress OR resilien* OR fatigue).ab
3. physician impairment/ OR burnout, professional/
4. physicians/ OR physicians, psychology/ OR physicians, family/ OR physicians, women
5. 1 OR 4
6. 2 OR 3
7. 6 AND 7
8. limit 8 to English language

#### Search strategy for CINAHL

1. (intern OR interns OR internship OR resident OR residents OR physician OR “medic* practi*” OR doctor OR surgeon OR registrar).ab
2. (burnout OR stress OR resilien* OR fatigue).ab
3. physicians/ OR surgeons/ OR anaesthesiologists/ OR foreign medical graduates/ OR geriatricians/ OR nephrologists/ OR paediatricians/ OR physician executives/ OR physicians, emergency/ OR physicians, family/ OR physicians, sports team/ OR physicians, women/ OR psychiatrists/ OR radiologists/ OR podiatrists/
4. burnout, professional/ OR stress, occupational/
5. 1 OR 3
6. 2 OR 4
7. 5 AND 6
8. limit 7 to English language

#### Search strategy for ProQuest

1. (intern OR interns OR internship OR resident OR residents OR physician OR “medic* practi*” OR doctor OR surgeon OR registrar).ab
2. (burnout OR stress OR resilien* OR fatigue).ab
3. 1 AND 2

## Additional file

Additional file 1: PRISMA checklist, file contains the completed PRISMA checklist for the systematic review. (DOC 65 kb)

## Abbreviations

CASP: Critical Appraisal Skills Programme
CBT: Cognitive Behavioural Therapy
CD-RISC: Connor-Davidson Resilience Scale
DP: Depersonalisation subscale of MBI
DV: Dependent variable
EE: Emotional exhaustion subscale of MBI
ESSI: Stress System Instrument
GHQ-12: General Health Questionnaire
ITT: Intention to treat
MBI: Maslach Burnout Inventory
OSATS: Objective Structured Assessment of Technical Skill
PA: Personal accomplishment subscale of MBI
PRISMA: Preferred Reporting Items for Systematic Reviews
PSS: Perceived Stress Scale
RCT: Randomised controlled trial
SORT: Strength of Recommendation Taxonomy
STAI: State-Trait Anxiety Inventory
